# 

**Published:** 1871-12

**Authors:** 


*American Practitioner.*

### Wanted.

Complete files of the former Buffalo *Medical Journal*, and volumes I, II and V of the present Buffalo *Medical and Surgical Journal*. Any one having the above journals *complete* can obtain satisfactory prices for them by addressing editor Buffalo *Medical and Surgical Journal.*

### Books and Pamphlets Received.

Elegant bound volumes of The American Practitioner, Vols. III and IV., David W. Yandell, M. D., and Theophilus Parvin, Editors.

Transactions of the Twenty-sixth Annual Meeting of the Ohio State Medical Society. Held at Cincinnati, April 4, 5 and 6, 1871. Cincinnati: Bosworth, Chase & Hall, 1871.

The Physicians' Daily Pocket Record, comprising a Visiting List, many useful memoranda, tables, etc., for 1872. Philadelphia, S. W Butler, M. D., 1872.

Modern Medical Therapeutics; a compendium of recent formulæ and specific Therapeutical Directions. By Geo. H. Napheys, A. M,, M. D. Third edition—revised and improved. Philadelphia, S. W. Butler, M. D., 1871.

Transactions of the American Medical Association, Vol. XXII, 1871.

Stimulants and Narcotics; Medically, Philosophically, and Morally considered. By Geo. M. Beard, M. D. New York, G. P. Putnam & Sons, 1871.

Boylston Prize Essay, 1871. Diseases of the Skin; the recent advances in their pathology and treatment. By B. Joy Jeffries, A. M., M. D. Reprinted from The American Journal of Syphilography and Dermatology. Boston, Alexander Moore, 1871.

Transactions of the Medical Society of the State of Pennsylvania at its Twenty-second Annual Session. Held at Williamsport, June, 1871.

The Principles and Practice or Surgery. By John Ashhurst, Jr., M. D. Illustrated with five hundred and thirty-three engravings on wood. Philadelphia, Henry C. Lea, 1871. Buffalo, Breed, Lent & Co.

On the Treatment of Pulmonary Consumption by hygiene, climate and medicine, in its connection with modern doctrines. By James Henry Bennet, M. D. Second edition. New York, D. Appleton & Co., 1872. Buffalo, Breed, Lent & Co.

Neuralgia and the diseases that resemble it. By Francis E. Austin, M. D., Lond. New York, D. Appleton & Co., 1872. Buffalo, Breed, Lent & Co.

Transactions of the American Ophthalmological Society, Eighth Anuual Meeting. Newport, July, 1871. New York, D. Appleton & Co.

Medical Thermometry, and Human Temperature. By C. A. Wunderlich and Edward Seguin, M. D. New York, Wm. Wood & Co., 1871. Buffalo, Breed, Lent & Co.

A Practical Treatise on Bright's Disease of the Kidneys. By T. Grainger Stewart, M. D. Second edition. New York, Wm. Wood & Co., 1871. Buffalo, Breed, Lent & Co.

A Handbook of Therapeutics. By Sidney Ringer, M. D. New York, Wm. Wood & Co., 1871. Buffalo, Breed, Lent & Co.

**Medical and Surgical Journal**

PUBLISHED MONTHLY,

Containing Original Articles, Reports of Medical Societies and Hospitals. Editorial, Reviews. Correspondence, Army News, etc., etc ; including the usual variety of Medical Periodical Publications. Specimen copies sent on application.

Address, Buffalo Medical & Surgical Journal, Buffalo, N. Y.

TERMS OF SUBSCRIPTION, - - $3.00 PER YEAR, IN ADVANCE.

